# CO_2_-induced climate change assessment for the extreme 2022 Pakistan rainfall using seasonal forecasts

**DOI:** 10.1038/s41612-025-01136-3

**Published:** 2025-07-09

**Authors:** Antje Weisheimer, Tim N. Palmer, Nicholas J. Leach, Myles R. Allen, Christopher D. Roberts, Muhammad Adnan Abid

**Affiliations:** 1 National Centre for Atmospheric Science (NCAS), Department of Physics, University of Oxford, Oxford, United Kingdom; 2 ECMWF, Reading, United Kingdom; 3 Department of Physics, University of Oxford, Oxford, United Kingdom

**Keywords:** Climate change, Atmospheric science

## Abstract

While it is widely believed that the intense rainfall in summer 2022 over Pakistan was substantially exacerbated by anthropogenic climate change^1,2^, climate models struggled to confirm this^3,4^. Using a high-resolution operational seasonal forecasting system that successfully predicted the extreme wet conditions, we perform counterfactual experiments simulating pre-industrial and future conditions. Both experiments also exhibit strong anomalous rainfall, indicating a limited role of CO_2_-induced forcing. We attribute 10% of the total rainfall to historical increases in CO_2_ and ocean temperature. However, further increases in the future suggest a weak mean precipitation reduction but with increased variability. By decomposing rainfall and large-scale circulation into CO_2_ and SST-related signals, we illustrate a tendency for these signals to compensate each other in future scenarios. This suggests that historical CO_2_ impacts may not reliably predict future responses. Accurately capturing local dynamics is therefore essential for regional climate adaptation planning and for informing loss and damage discussions.

## Introduction

Recent attempts to estimate the role that anthropogenic climate change played in the development of the extreme seasonal rainfall, and subsequent devastating flooding, during the summer 2022 over Pakistan were not successful because state-of-the-art climate models struggled to simulate the essential rainfall characteristics of the South Asian summer monsoon^3–6^. In particular, these attempts concluded that the current generation of climate models is unable to provide a basis to confidently quantify the monsoon season rainfall intensity with climate change and thus the extent to which the summer 2022 Pakistan flooding was exacerbated by climate change.

**Fig. 1:**
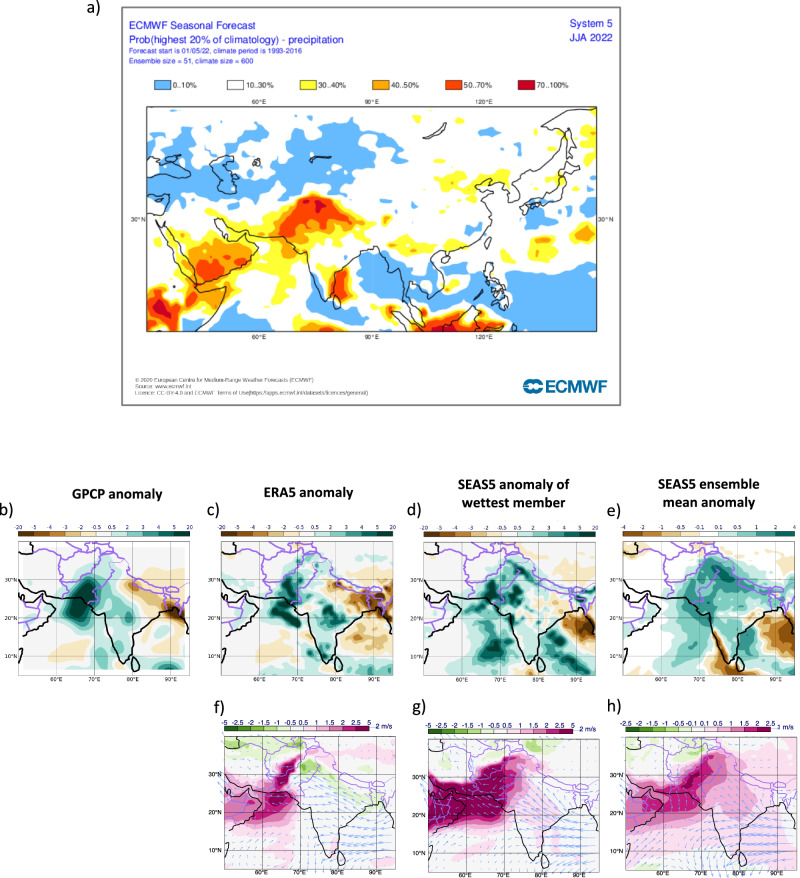
Seasonal forecasts and observations for the summer 2022. **a** Operational SEAS5 forecast for JJA 2022 issued in May 2022 of the probability that precipitation will exceed the 80th climatological percentile (upper quintile), highlighting a 50-70% and more probability over Pakistan. *© [2022] European Centre for Medium-Range Weather Forecasts (ECMWF*). Precipitation anomalies in JJA 2022 for GPCP (**b**), ERA5 (**c**), the wettest ensemble member (**d**) and the SEAS5 ensemble mean (**e**). Specific humidity (colour shades) and wind at 850 hPa for ERA5 (**f**), the wettest ensemble member (**g**) and the SEAS5 ensemble mean (**h**). All anomalies are computed with respect to their corresponding climatologies over the reference period 1993–2016. Precipitation units: mm/day, specific humidity units: g/kg and wind units: m/s.

Here we study this question from a different perspective. We have argued elsewhere how seasonal forecasts can provide valuable information about the trustworthiness of the climate system’s response to forcing^7^—information that past and current generation climate models are not able to provide^5,8–13^. We use state-of-the-art seasonal predictions performed with the operational seasonal forecasting system SEAS5^14^ of the European Centre for Medium-Range Weather Forecasts (ECMWF) at a higher resolution than most CMIP6 climate models (see Methods). The seasonal forecasts have demonstrated^14,15^ significant skill in past predictions of June-August (JJA) precipitation over Pakistan with historical correlations of around 0.5 between the SEAS5 ensemble mean forecasts and observations from GPCP^16^. SEAS5 indeed showed a very strong precipitation signal in their forecast for JJA 2022 issued in May 2022: the probability of exceeding the climatological 80^th^ rainfall percentile, or upper quintile, was raised from historically 20% to 50–70% and beyond (Fig. 1a). It is worth noting that other similar operational prediction models that contribute to the Copernicus Climate Change C3S multi-model ensemble of seasonal forecasts also predicted strongly enhanced rainfall for Pakistan for JJA 2022^17,18^. The successful predictions of the extreme event in question together with the high confidence in the forecast outcome based on past performance suggest that our forecast-based approach^19,20^ could be valuable in attributing and quantifying the role of anthropogenic climate change for the extremely wet summer 2022 season in Pakistan.

## Results

### Anomalous conditions in summer 2022

Pakistan’s location in the transition zone between the moist South Asian monsoon climate and the arid hot climate of Southwest Asia makes it susceptible to influences from both^21^. The Asian Summer Monsoon with its seasonally reversing winds and corresponding changes in precipitation is one of the most important weather and climate phenomena for the Indian subcontinent and surrounding regions. It affects Pakistan primarily via two distinct routes: south-easterly winds from the Bay of Bengal bringing air along the foothills of the Himalayas to reach Pakistan’s northern provinces, and south-westerly airmasses from the Arabian Sea, advecting moisture into the southern provinces^22^.

One of the defining characteristics of the climate of the Indian subcontinent is its large interannual rainfall variability, often attributed to large-scale sea surface temperature (SST) anomalies associated with El Niño Southern Oscillation (ENSO) and the Indian Ocean Dipole (IOD) but also to factors including the springtime snow depth in the Himalayas, the presence of aerosols^23,24^ and internal dynamics of subseasonal variations of active - break periods^25^.

**Fig. 2:**
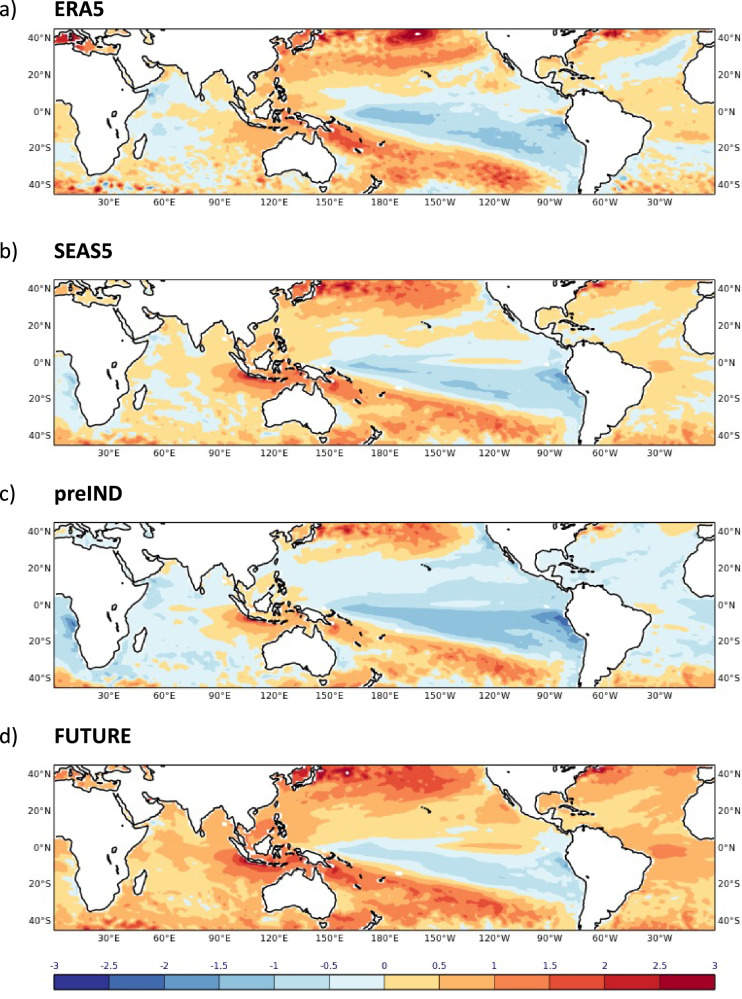
SST anomalies in JJA 2022. Maps show the anomalies for ERA5 (**a**), SEAS5 (**b**), preIND (**c**) and FUTURE (**d**). Anomalies in **c**, **d** are computed with respect to the SEAS5 hindcast climatology. SST units: K.

GPCP^16^ and ERA5^26^ reanalysis data for JJA 2022 (Fig. 1b, c) clearly show the enhanced precipitation over Pakistan with maxima of anomalous rainfall over the central and southern provinces. GPCP data are available on a 2.5 degree horizontal grid while ERA5 and all model data are interpolated on a 1 degree grid. By contrast, the monsoon circulation over India and the Bay of Bengal weakened with dry anomalies over north-east India and Bangladesh. Large-scale south-easterly wind anomalies at 850 hPa extend from that region into Pakistan and reduce the flow of moist air across much of Northern India. This anomalous circulation pattern was previously identified as a principal mode of variability associated with enhanced rainfall over Pakistan^27,28^. A further relevant characteristic of the local atmospheric circulation in the summer 2022 is the anomalously moist air over the Arabian Sea off the coast of Pakistan which is transported inland with the mean monsoon flow. The tropical SSTs in JJA 2022 are dominated by cold anomalies of the La Niña phase of ENSO in the equatorial central and east Pacific and a negative IOD with colder temperature anomalies in the western Arabian Sea relative to the eastern Indian Ocean (Fig. 2a).

Amid the anomalous meteorological conditions in southern Asia during the summer 2022, it is important to note that whilst Pakistan to the south-west of the Tibetan Plateau had an extremely wet and cold season, the Yangtze River Valley east of the Tibetan Plateau experienced unprecedented heat^29–31^. The anomalous upper tropospheric zonal flow over Tibet is dynamically linked to opposite vertical atmospheric motion at either sides of the plateau which explains a major fraction of these two contrasting extreme events^29,31^. We will come back to analyse the significant role of this large-scale flow anomaly in shaping the precipitation response over Pakistan, including under climate change, in the sub-section on the role of the upper tropospheric zonal winds.

### Seasonal forecasts of JJA 2022 over Pakistan

The spatial distribution of the ensemble-mean precipitation anomalies in the SEAS5 forecasts (Fig. 1e) shows widespread areas of Pakistan predicted to receive anomalous rainfall. The Pakistan-average ensemble-mean absolute precipitation rate of 2.71 mm/day is higher than in any other JJA season of the retrospective hindcast period 1993–2016 for which the SEAS5 system was tested, being nearly 3 standard deviations above the hindcast period mean. This amounts to an ensemble-mean anomaly of 0.88 mm/day or approximately 150% of the SEAS5 climatological rainfall during the hindcast reference period. The probability of exceeding the upper quintile of the climatological rainfall distribution during JJA 2022 is 73% for the country-wide average, which is notably much larger than its climatological value of 20%.

For comparison, the ERA5 absolute rainfall average over Pakistan during JJA 2022 amounts to 4.20 mm/day, corresponding to approximately 3 standard deviations above the hindcast mean, an anomaly of 2.1 mm/day and 200% of its climatological mean, and substantially exceeding any other year’s amount over the similar historical period. GPCP’s absolute rainfall over Pakistan amounts to 4.09 mm/day, which corresponds to 3.5 standard deviations, an anomaly of 2.3 mm/day and approximately 230% of its climatological mean. In the following, we restrict our comparison mainly to ERA5, acknowledging that GPCP data are broadly similar in spatial structure and anomalous amount.

The reason for the smaller SEAS5 ensemble-mean rainfall amount when compared to the observation-based reanalysis is simple—the ensemble mean forecast is a smoothed estimate based on 51 individual forecast member realisations from which the unpredictable “noise” amongst the members has been filtered. It is important to note though that the wettest ensemble member (Pakistan-wide: 4.31 mm/day), shown in Fig. 1d, is indeed able to produce a similar magnitude of total rainfall to those in ERA5, highlighting the realism of the SEAS5 coupled ocean-atmosphere model. Since coarser-scale climate models notoriously underestimate rainfall extremes, these results demonstrate a remarkable success in modelling extreme seasonal-mean precipitation amounts and increase the trustworthiness of these seasonal forecasts.

The extreme rainfall anomalies in the mean forecast are associated with increased lower tropospheric specific humidity and a weakened 850 hPa monsoon circulation across the Indian subcontinent, see Fig. 1f, and reflect the observed situation accurately. In particular, the south-easterly flow anomaly from the Bay of Bengal across northern parts of India is well captured. The wettest ensemble member is associated with a strong positive humidity anomaly over the Arabian Sea and Pakistan and strong south-easterly wind anomalies over the sea off the coast of Pakistan (Fig. 1g). The ensemble member with the strongest rainfall over Pakistan also predicts the largest specific humidity anomaly across the SEAS5 ensemble in the region and shows the second strongest easterly anomaly in the winds within the ensemble across the Pakistan coast. The level of extremeness of the humidity anomaly reaches nearly 3 standard deviations of the model climatology, whereas the wind anomaly is less extreme with approximately 1.5 standard deviations. Overall, these results confirm the important roles of both the humidity and the atmospheric circulation for simulating realistic rainfall totals.

The tropical SST conditions with cold La Niña temperatures over the Pacific and a negative dipole over the Indian Ocean are well reproduced in the SEAS5 forecasts (Fig. 2b).

### Climate change experiments for the summer 2022

To estimate the extent of loss and damage associated with the extreme rainfall and caused by human-induced climate change, we must first be able to quantify reliably by how much the JJA 2022 rainfall anomalies over Pakistan were exacerbated by climate change. However, as noted above, conventional climate models are unable to simulate important rainfall characteristics like the magnitude of precipitation extremes, thus rendering any derived quantification of the role of climate change unreliable. On the other hand, we have shown that the ECMWF high-resolution SEAS5 model had forecast this event with good fidelity. We thus use the successful simulations of SEAS5 as a starting point for our investigations into the role of anthropogenic CO_2_-driven climate change on the extreme rainfall. By only modifying the radiative forcing of the control forecasts together with perturbing the ocean initial conditions correspondingly, we will show how the SEAS5 anomalies respond to these perturbations, while all other conditions are kept unchanged. Such a reductionist approach provides insight into the specific roles of the imposed changes, while avoiding the inclusion of additional confounding factors.

In particular, we create two additional seasonal forecasts for JJA 2022 in hypothetical, or counterfactual, worlds: the first (“preIND”) uses atmospheric CO_2_ concentrations that are fixed at pre-industrial levels of 285 ppm, and in the second (“FUTURE”) the CO_2_ concentrations were increased to 615 ppm^19,20^. Present-day concentrations of 415 ppm are used in operational SEAS5 forecasts. In the forecast experiments for these counterfactual worlds, we not only change the CO_2_ concentrations, but also adjust the forecast initial conditions to account for past and future warming. Since seasonal predictability is primarily accounted for by information in ocean initial conditions^32^, for the counterfactual forecasts we have adapted the ocean initial conditions such as to reflect the impact of CO_2_ concentrations on the initial state of the ocean. Details of the technique to perturb the ocean, together with a description of all model experiments, are given in the Methods Section.

**Fig. 3:**
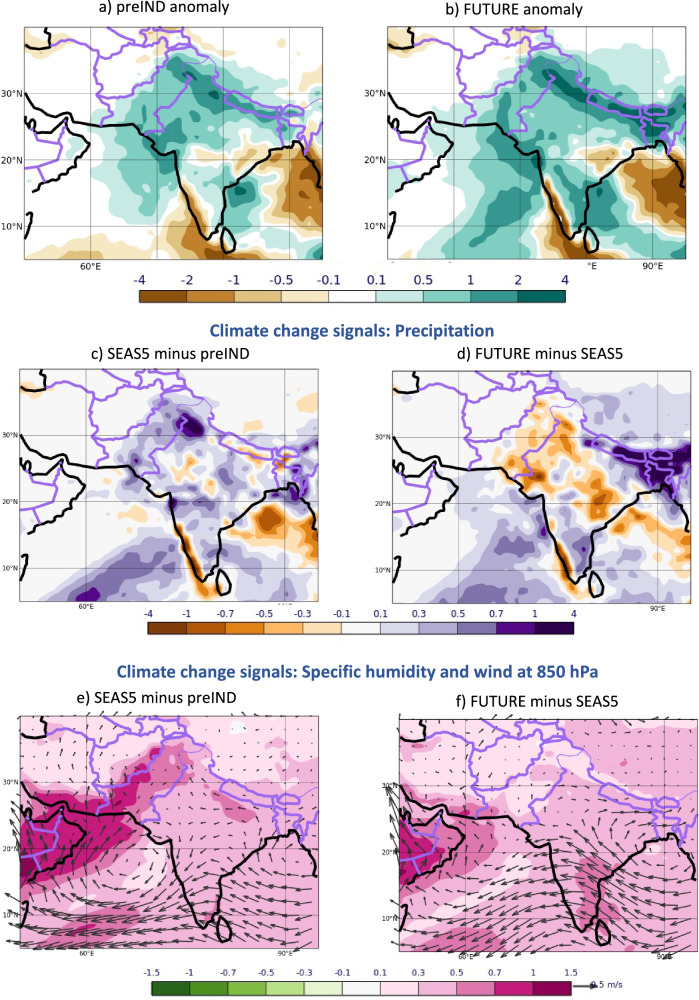
Precipitation in the counterfactual experiments and climate change signals for precipitation, specific humidity and winds at 850 hPa. Precipitation anomalies in the preIND (**a**) and FUTURE (**b**) simulations. **c–f** Climate change signals of JJA 2022 in the past (**c**, **e**) and for the future (**d**, **f**) for precipitation (**c**, **d**) and specific humidity with winds at 850 hPa (**e**, **f**). Climate change signals are defined as the difference between simulations with stronger CO_2_ and SST forcings minus simulations with weaker CO_2_ and SST forcings. The past climate change signals (**c**, **e**) are estimated as SEAS5 minus preIND; the future climate change signals (**d**, **f**) are estimated from FUTURE minus SEAS5. Precipitation units: mm/day, specific humidity units: g/kg and wind units: m/s.

The JJA 2022 seasonal-mean ensemble-mean rainfall anomalies and climate change signals from both counterfactual forecasts preIND and FUTURE are displayed in Fig. 3a, b. Here, the past signal is estimated as SEAS5 minus preIND and the future signal as FUTURE minus SEAS5, so that both signals reflect the impact of the increased forcing. The rainfall anomalies over Pakistan in both experiments are large, though smaller (0.60 mm/day in preIND and 0.75 mm/day in FUTURE) than in SEAS5 (0.88 mm/day, see Fig. 1e), which shows that the magnitude of the rainfall event of the past and future simulations is similar to present-day conditions and implies that neither counterfactual has a strong impact on the extreme wet conditions per se. Even under preindustrial or strongly increased CO_2_ forcing levels in the future, the summer in Pakistan would have been extremely wet. This indicates a limited role of the CO_2_ forcing for the extreme event.

The historical rainfall climate change signal, that is the difference between the operational forecast of SEAS5 and the experiment preIND, shows a small increase of the ensemble-mean precipitation over Pakistan in SEAS5 compared to what it would have been if CO_2_ concentrations in JJA 2022 were at pre-industrial levels and the global oceans were colder (Fig. 3c). For the country-wide average, the counterfactual preIND prediction of 2.43 mm/day amounts to a 0.28 mm/day, or approximately 10%, reduction over the current-day SEAS5 forecast. This mean rainfall change from preIND to SEAS5 is statistically significant with a *p*-value of 0.015. The past climate change signals of specific humidity and winds at 850 hPa (Fig. 3e) indicate strengthened monsoon winds leading to an increased south-westerly transport of moister air from the Arabian Sea into Pakistan, contributing to the increased precipitation signal.

The mean climate change signal of precipitation for future conditions (Fig. 3d), that is the difference between the FUTURE forecast experiment and the SEAS5 forecasts, changes its sign: further increased CO_2_ concentrations and warming of the global oceans do not further increase the mean precipitation totals over Pakistan as they did in the past. The FUTURE ensemble-mean rain is reduced over the operational SEAS5 forecast by 0.13 mm/day to 2.58 mm/day. If the response to a further rise of CO_2_ were purely thermodynamic, a further increase in mean rainfall in FUTURE would have been expected. However, FUTURE shows a (non-significant) reduction of approximately 5% in Pakistan-wide rainfall over the SEAS5 forecasts instead.

The associated lower tropospheric humidity and circulation signals in FUTURE (Fig. 3f) are consistent with the precipitation signal. Humidity increases between SEAS5 and FUTURE, especially over the Arabian Sea, are reduced compared to the past signal between preIND and SEAS5. The very weak response from SEAS5 to FUTURE in winds off the coast of Pakistan suggests that the increased advection of moist air from the sea into Pakistan, as found in the past, is absent in the future climate change signal.

### Characteristics of the rainfall distributions over Pakistan

**Fig. 4:**
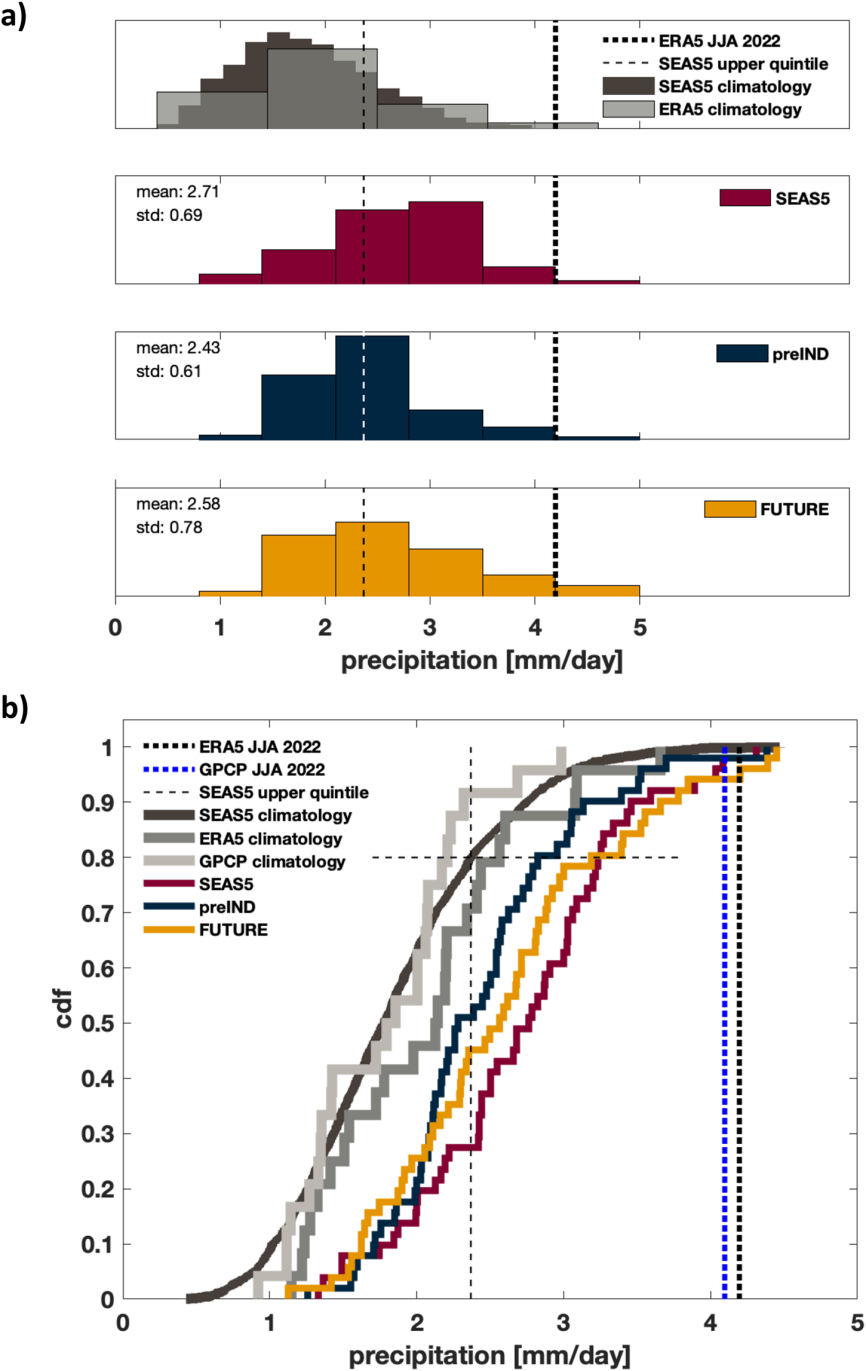
Distributions of the rainfall over Pakistan in the different datasets and experiments. **a** Histograms with the light grey showing JJA precipitation in ERA5 over the 24-year climatological reference period 1993–2016. The dark grey histograms shows JJA precipitation in SEAS5 over the same period estimated form all 51 hindcast ensemble members. Forecast distributions for JJA 2022 from the 51 SEAS5 (red), preIND (dark blue) and FUTURE (yellow) ensemble members. The dotted vertical lines in all panels indicate the amount of rainfall during JJA 2022 in ERA5 and GPCP. The dashed vertical line in all panels indicates the rainfall threshold of the upper quintile of the SEAS5 climatological distribution. The inset numbers indicate the ensemble mean and standard deviation for each forecast ensemble. All histograms have been normalised such that the sum of the bar areas equals 1. **b** Cumulative distributions of precipitation in ERA5, GPCP and for the different simulations.

It is, of course, important to examine not just the ensemble mean but also the full ensemble distributions of rainfall. To this end, we show histograms (Fig. 4a) and cumulative distribution plots (Fig. 4b) of JJA absolute rainfall averaged over Pakistan for the SEAS5 seasonal forecasts and the two counterfactual forecasts preIND and FUTURE. The climatological distributions of ERA5, GPCP and SEAS5 during the 1993–2016 period are displayed in Fig. 4 in different shades of grey colour. The climatological distribution of SEAS5 is based on 51 ensemble members in the hindcasts, resulting in a much finer histogram and smoother distributions than the observed datasets. There is a remarkably good agreement between the model climatology and the ERA5/GPCP distributions, with comparable levels of variability and only a very small dry bias in the hindcasts.

With the 51-member operational SEAS5 forecast plotted in red, both the histograms and cumulative distributions highlight the shift to extremely wet conditions in JJA 2022 compared to the SEAS5 hindcast climatology. This shift towards higher rainfall impacts not only the mean but the entire forecast distribution including the dry and wet extremes. From Fig. 4b we see that the climatological SEAS5 upper quintile threshold of 2.37 mm/day (i.e., 80th climatological percentile of the seasonal mean rainfall distribution, see dashed vertical line) is exceeded by approximately 70% of the SEAS5 forecast ensemble members. The wettest forecast member produces slightly more rain than ERA5 and GPCP.

The distributions of both counterfactual ensembles, shown in Fig. 4 in dark blue for the preIND reduced CO_2_ concentration experiment and in yellow for the FUTURE increased CO_2_ concentration experiment, are also clearly shifted towards wetter-than-normal conditions, similarly to SEAS5. By taking the quantile of the cumulative forecast distributions in Fig. 4b at the climatological upper quintile precipitation threshold, we estimate that the probability of exceeding the climatological SEAS5 upper quintile in preIND is nearly 50% which implies that the risk of high precipitation amounts is still greatly increased over its reference climatological value of 20%, yet not as much as in the SEAS5 forecast using current-day CO_2_ concentrations. Equally, the FUTURE probability of exceeding the upper quintile, 55%, remains strongly enhanced over its climatological reference, though less so than in SEAS5.

This significant and consistent shift away from the climatology towards very wet conditions implies that other factors not directly influenced by the CO_2_ concentrations and global ocean temperatures were mainly responsible for the anomalous rainfall in the summer 2022 forecasts. Tropical SST anomalies in both the preIND and FUTURE experiments show clear signals of La Niña and a negative IOD (Fig. 2c, d). However, precipitation over south-west Asia is highly variable on interannual timescales^21^. While intermittent co-occurrences of ENSO events during extreme summer rainfall seasons over Pakistan were observed in the past, the overall statistical relationship of ENSO-forced teleconnections is weak and not robust. Dynamical processes of internal atmospheric variability like jet stream meandering and circumglobal wave patterns are known to modulate the precipitation distribution over the region. The simultaneous acting of several oceanic and atmospheric drivers can lead to amplified precipitation responses^19^. An in-depth analysis of the physical nature of these potential drivers is not the focus of this study and has been discussed elsewhere^18,29–31^.

In summary, we find that the overall shift towards very wet absolute conditions for the 2022 summer season is still observed in both counterfactual experiments, highlighting the primary role of other drivers than anthropogenically induced CO_2_ for interannual rainfall variability and thus the extreme event of 2022. In addition, both perturbed forecast ensembles produce less mean rainfall than the operational SEAS5 forecast. The cumulative distribution plots in Fig. 4b clearly indicate the reduction in rainfall for the counterfactual experiments across the distributions. While the upper tail of the preIND distribution remains drier than SEAS5, the FUTURE ensemble includes several extreme members with more rainfall than the wettest members in SEAS5 and 3 ensemble members exceeding the ERA5 rainfall totals for 2022. This increase in variability for the FUTURE simulation is, however, too small to prove statistically significant in an *F*-test.

### The role of the upper-troposphere zonal wind

How can our rainfall results - where past climate change from pre-industrial conditions to today *increased* the precipitation over Pakistan by up to 10%, yet similar forcings in the future tend to slightly *decrease* it - be understood?

An important aspect to the extreme wet season in Pakistan is the anomalous zonal flow over the Tibetan Plateau. In summer, the climatological high potential temperatures over the western Tibetan Plateau together with the climatological upper-tropospheric subtropical westerly flow generate descending vertical motion to the west and ascent further east. Climatological descent to the west is associated with warming and drying and has a direct effect on maximum temperatures and precipitation at the surface^29^. On the downstream eastern side of the plateau, the ascending vertical motion leads to a wetter climate over sub-tropical East Asia.

**Fig. 5:**
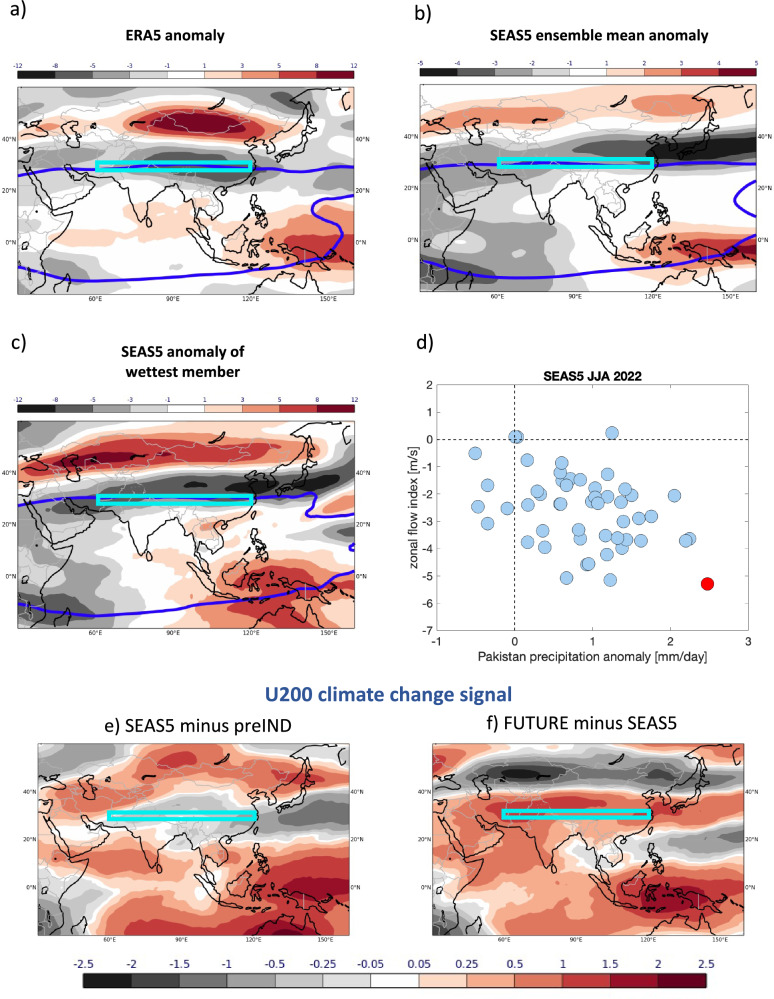
Upper tropospheric circulation. Anomaly of the zonal wind at 200 hPa (U200) during JJA 2022 in (**a**) ERA5, (**b**) SEAS5 ensemble mean and (**c**) the ensemble member with the strongest precipitation anomaly over Pakistan. The blue thick lines indicate the reversal of tropical easterlies to extratropical westerly flow. The cyan coloured boxes in (**a**–**c**) show the area over the Tibetan Plateau that is used to define the zonal flow index. **d** Scatter diagram of the relationship between the zonal flow index and precipitation anomaly over Pakistan in the SEAS5 forecast ensemble. The ensemble member with the highest precipitation anomaly is highlighted. U200 climate change signals of JJA 2022 in the past (**e**) and for the future (**f**). Climate change signals are defined as the difference between simulations with stronger CO_2_ and SST forcings minus simulations with weaker CO_2_ and SST forcings. The past climate change signal (**e**) is estimated as SEAS5 minus preIND; the future climate change signal (**f**) is estimated from FUTURE minus SEAS5. Wind units: m/s.

In JJA 2022, however, the westerly wind circulation in the upper troposphere over Tibet and surrounding areas was substantially weaker than in its long-term climatological mean^29^. The resulting negative zonal wind anomalies at 200 hPa in ERA5 are shown in Fig. 5a. With the blue line indicating the region of easterly upper tropospheric winds in the tropics, these anomalies can be interpreted as an expansion of the tropical easterly regime and a northward shift of the westerly jet. The SEAS5 forecasts correctly predicted large-scale easterly anomalies over subtropical Asia and the Tibetan Plateau (Fig. 5b), yet differences of zonal flow anomalies exist across the ensemble, manifesting in a weaker ensemble mean circulation anomaly locally over Pakistan and an eastward shift of the maxima of the circulation anomalies.

Based on reanalysis and observational data, it has been demonstrated elsewhere^29^ that the easterly flow anomalies in the upper troposphere over the Tibetan Plateau during the summer 2022 dynamically induced strong ascent to the west of the plateau, leading to the observed positive rainfall anomalies in Pakistan. To test whether a similar dynamic mechanism is active in our forecasts, we quantify the relationship between the large-scale upper tropospheric circulation and rainfall over Pakistan by defining a zonal flow index as the mean zonal wind anomaly at 200 hPa over a region above the Tibetan Plateau where the strongest easterly wind anomalies occurred (cyan box in Fig. 5a, see Methods). The zonal flow index of the ERA5 anomaly is -5.1 m/s, for the ensemble mean of SEAS5 it is -2.6 m/s with the largest negative anomaly within the ensemble being -5.3 m/s.

The scatterplot of the SEAS5 zonal flow index anomalies for each ensemble member against its rainfall anomaly in Pakistan in Fig. 5d clearly shows that our model captures the described observed relationship between the large-scale circulation anomaly and rainfall over Pakistan, with the correlation of *r* = *-0.39* being highly significant (*p* = *0.004*). Indeed, the ensemble member with the highest rainfall (see also Fig. 1d) also shows the strongest upper air easterly wind anomaly (Fig. 5c).

Given the realism of the physical mechanism connecting atmospheric circulation anomalies with precipitation signals in Pakistan in the SEAS5 model, the question arises whether the sign change of the precipitation climate signal from pre-industrial to future conditions (Fig. 3c, d), can also be detected in the large-scale upper-air zonal flow climate signals. In Fig. 5e, f we see indeed that in the past the total climate change signal showed a mean weakening of the westerly zonal flow component by approximately 0.3 m/s (Fig. 5e), consistent with a weak increase in precipitation. In contrast, the future climate change signal shows a mean strengthening of the westerly flow component by approximately 0.9 m/s (Fig. 5f), consistent with the weak decrease in precipitation found in the simulations. The statistical relationship between the ensemble rainfall anomalies over Pakistan and the upper tropospheric zonal flow index is slightly reduced in the preIND counterfactual simulation (*r* = *-0.34* with *p* = *0.015*) and slightly strengthened in the FUTURE simulation (*r* = *-0.46* with *p* = *0.001*). As with the precipitation sensitivities, the circulation signals induced by changes in the CO_2_ and SST forcings are small compared to the magnitude of the upper tropospheric wind anomaly (approximately 10% and 30%).

### The direct CO_2_ and SST contributions to the climate change signal

While these findings for the upper tropospheric circulation changes are in full agreement with the results for precipitation, they alone cannot tell us what causes the opposing signals in the past and future. We have thus performed two additional numerical experiments, A and B, to help us disentangle the contributions behind the change in the climate signals. The motivation for these experiments is the following: Our hypothetical climate change experiments both apply two simultaneous perturbations to the physical climate system: to the CO_2_ forcing in the atmosphere and to the initial 3D-state of the ocean temperatures as felt by the atmosphere through the SSTs. From these it remains unclear how each of these two perturbations individually impact the precipitation signal over Pakistan. Difference in their individual contributions in the past and future can potentially lead to the nonlinear behaviour that we find for the precipitation climate change signal.

**Fig. 6:**
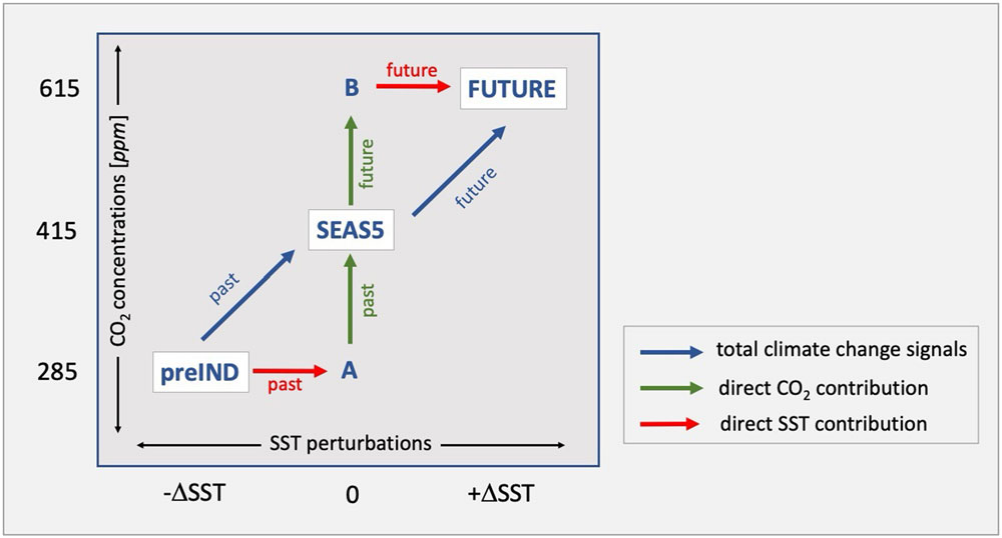
Schematic illustrating the design of the forecast attribution experiments and the direct atmospheric contributions from CO_2_ and SST perturbations to the total climate changes signals. The operational forecast based on SEAS5 uses CO_2_ concentrations of 415 ppm and serves as the reference. Contrasting SEAS5 with preIND defines the past total climate change signal; contrasting FUTURE with SEAS5 determines the future total climate change signal. The two additional experiments A and B use lower and higher CO_2_ concentrations than SEAS5 without simultaneously perturbing the SSTs and thus allow to estimate the direct CO_2_ and SST contributions to the total climate change signals, as indicated by the green and red arrows.

The schematic in Fig. 6 illustrates our approach, how the additional experiments A and B complement the preIND and FUTURE experiments, and what they can tell us about the relative roles of the CO_2_ and SST perturbations. Experiment A uses pre-industrial CO_2_ concentrations while the SSTs and 3D ocean and sea-ice temperatures (at initial time) are the same as in SEAS5. Contrasting A with SEAS5 reveals the past effect that perturbations of the CO_2_ forcing had without accounting for changes in the ocean (hereafter called past direct CO_2_ contribution). Contrasting A with preIND reveals the past effect from initial SST and sea-ice perturbations only, without accounting for changes in CO_2_ (hereafter called direct past SST contribution). By analogy, experiment B is forced with increased future CO_2_ concentrations while the initial SSTs are kept the same as in SEAS5. Here, the difference between B and SEAS5 reveals the future direct CO_2_ contribution and neglects changes in the ocean. The difference of FUTURE and B uncovers the corresponding future direct SST contribution while ignoring CO_2_ changes. The sum of the direct CO_2_ and direct SST contributions equals the total climate change signal that we discussed in Fig. 3c, d for precipitation and in Fig. 5e, f for the upper tropospheric zonal winds.

**Fig. 7:**
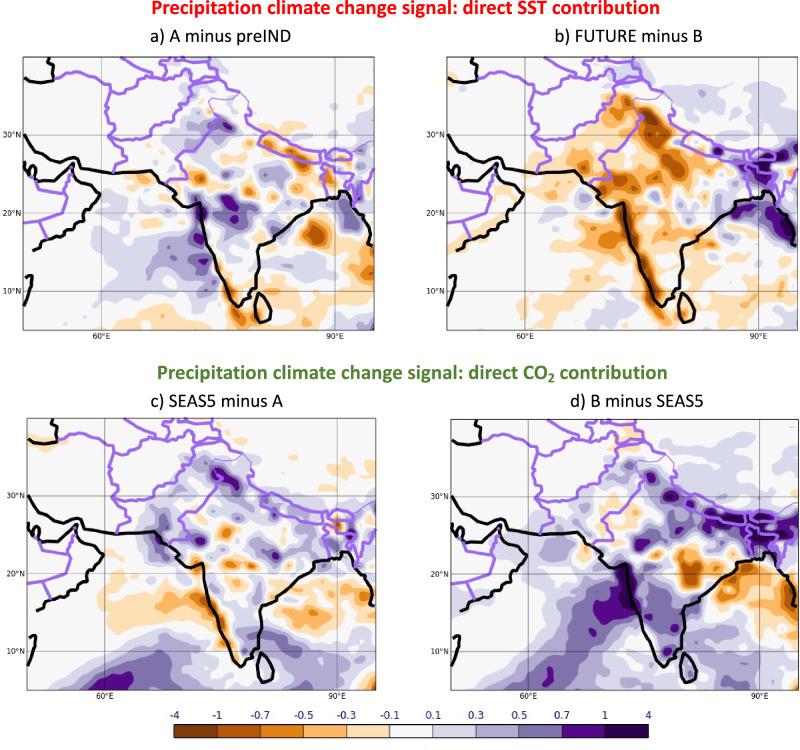
Contributions to the total precipitation climate change signals of JJA 2022 in the past (left) and for the future (right) directly from the SSTs (top) and CO_2_ (bottom). The total climate change signals (shown in in Fig. 3a, b) are the sum of contributions from direct SST and CO_2_ perturbations. All signals are defined as the difference between simulations with stronger SST/CO_2_ forcings minus simulations with weaker SST/CO_2_ forcings. Further illustration of all experiments is given in the schematic of Fig. 6. Precipitation units: mm/day. Past changes are shown on the left (**a**, **c**) and future changes on the right (**b**, **d**). SST changes are shown at the top (**a**, **b**) and CO_2_ changes are shown at the bottom (**c**, **d**).

**Fig. 8:**
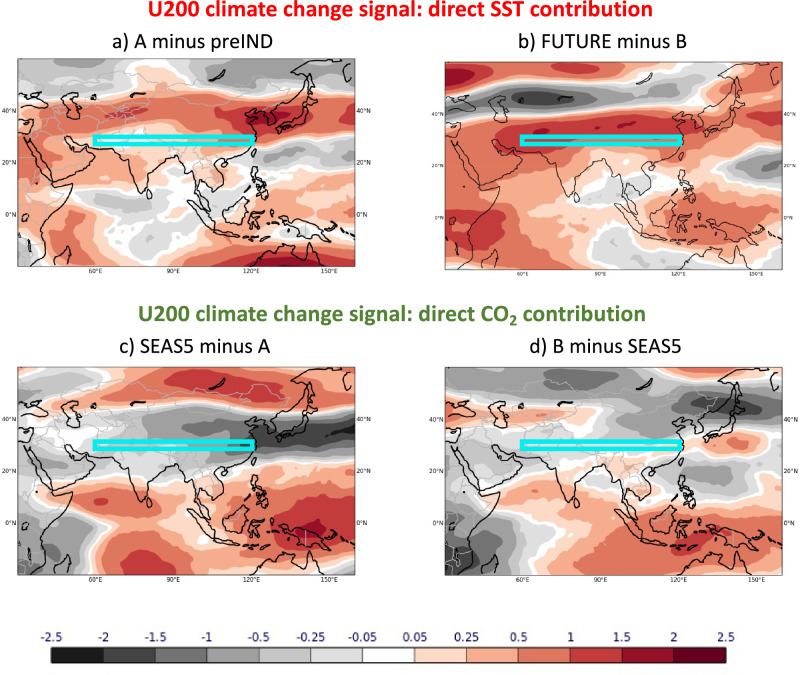
Contributions to the total zonal wind at 200 hPa (U200) climate change signal of JJA 2022 in the last and for the future directly from SSTs and CO_2_. Past changes are shown on the left (**a**, **c**) and future changes on the right (**b**, **d**). The total climate change signals (shown in Fig. 5e, f) are the sum of contributions from direct SST (**a**, **b**) and CO_2_ (**c**, **d**) perturbations. All signals are defined as the difference between simulations with stronger SST/CO_2_ forcings minus simulations with weaker SST/CO_2_ forcings. Further illustration of all experiments is given in the schematic of Fig. 6. Wind units: m/s.

Results from the direct contribution experiments are shown in Fig. 7 for precipitation and of Fig. 8 for the upper-tropospheric zonal winds. The direct SST contribution leads to a weak wet signal (0.08 mm/day) over Pakistan for past changes (Fig. 7a). The future direct SST contribution (Fig. 7b), however, reveals a drying signal of -0.25 mm/day if the ocean temperatures are further increased. In contrast, the direct CO_2_ contribution signal is estimated to be to consistent increased rainfall in the past (0.20 mm/day) and in the future (0.12 mm/day), see Fig. 7c, d. We can thus conclude that the CO_2_ perturbations primarily contribute to the total signal of increasing precipitation (Fig. 3c) due to climate change in the past. The situation in the future is different though: the drying due to warmer future SSTs overcompensates the continued wet signal from the direct CO_2_ contribution, resulting in a total dry signal for future climate change (Fig. 3d).

The dynamically linked upper air zonal flow over the Tibetan Plateau shows in Fig. 8 that the direct SST contributions consistently act to increase the westerly flow component in the past and future in the region of the zonal flow index (Fig. 8a, b) with increased forcing. The direct CO_2_ contributions, on the other hand, consistently act in the opposite direction and weaken the westerly (or increase the easterly) flow component (Fig. 8c, d). This consistency in the sign of the individual climate change circulation response to the CO_2_ and SST effects lends support to the robustness of the physical and dynamical processes involved in both.

However, despite this agreement in the individual responses, the total zonal wind signal in the future with its westerly flow component (Fig. 5f) is opposite to the easterly flow component of the past signal (Fig. 5e). Through the dynamical links described, these circulation changes can be understood as the result of a wet climate change signal for Pakistan in the past but a drying signal in the future. Our decomposition analysis reveals that the varying magnitudes, or strength, of the individual CO_2_ and SST direct effects, lead to the described non-linearities in the rainfall response to climate change. In the past, the direct CO_2_ contribution effect dominated the upper air circulation, and the combined effect of the SSTs and CO_2_ resulted in a strengthening of the easterly flow component over the Tibetan Plateau, and thus led to increased precipitation over Pakistan. The radiative effects of the CO_2_ changes alone (that is without their effect on the ocean temperatures) act to shift the westerly jet northwards and strengthen the upper tropospheric easterlies over the Tibetan Plateau, counteracting the direct SST effect to weaken the easterly anomalies. In the future, however, the SST effect of warmer ocean temperatures becomes stronger, relatively more important and outweighs the direct CO_2_ effect. The net result is a weakening of the easterly flow component (strengthening of the westerly component) and thus a reduction in rainfall over Pakistan.

**Fig. 9:**
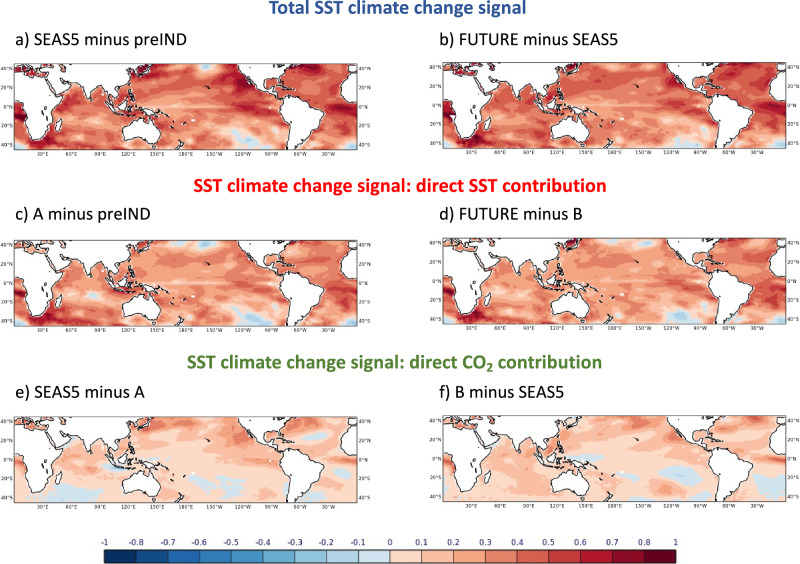
SST climate change signals of JJA 2022 in the past (left) and for the future (right). The total climate change signals (**a**, **b**) are the sum of contributions from direct SST (**c**, **d**) and CO_2_ (**e**, **f**) perturbations. All signals are defined as the difference between simulations with stronger CO_2_ and SST forcings minus simulations with weaker CO_2_ and SST forcings. Illustration of all experiments is given in the schematic of Fig. 6. Temperature units: K.

Since all forecasts are performed with a coupled atmosphere-ocean system, the ocean temperatures interact with the atmosphere over time. In the perturbed CO_2_ concentration-only experiments where the initial conditions of the ocean are unperturbed (Fig. 6a, b), the SSTs will start to adapt to the CO_2_ forcings and thus produce, in addition to the direct CO_2_ contributions, an indirect SST imprint on the climate change signals. As we saw in the analysis above, the effect of the SSTs works in the opposite direction to the effect of the CO_2_ concentrations. This negative feedback between the direct SST and CO_2_ effects means that the direct CO_2_ contributions shown in Figs. 7a, b and 8a, b are conservative estimates of the “true” individual contributions because of the slow indirect SST increases during the 4-month-long simulations. The “true” direct CO_2_ contributions if the SSTs would not contribute are thus likely larger than those shown in the figure. We estimate the indirect SST warming to be roughly 50% of the total SST change in our preIND and FUTURE experiments relative to SEAS5 (Fig. 9).

## Discussion

There are a number of important implications that can be drawn from this study. Relevant to the loss and damage issue, the results suggest that past anthropogenically caused climate change due to CO_2_ does exacerbate the rainfall amounts over Pakistan in summer 2022 by approximately 0.3 mm/day. In absolute terms, however, this increase is small: around 10% of the SEAS5-simulated absolute rainfall amount of 2.71 mm/day for JJA 2022. Contrary to a linear projection of this signal into the future, our experiments show that a future anthropogenic increase in CO_2_ concentrations would not necessarily lead to a further exacerbation of Pakistan rainfall; instead, a non-significant reduction by approximately 5% of the ensemble mean rainfall has been found.

The small magnitude of the attributable past climate change rainfall signal suggests that even under pre-industrial CO_2_ conditions, our seasonal prediction would have forecast an increased probability of exceptionally wet conditions. Other factors inherent in the state of the coupled climate system and its forcings which are important for interannual climate variability, such as the La Niña in the tropical Pacific, would have driven the forecast probabilities towards significantly wet conditions for Pakistan, outweighing the minor effect of changes to the CO_2_ concentrations.

We decompose the total CO_2_-induced climate change signal of rainfall in Pakistan into its effects due to the direct atmospheric response to CO_2_ and due to the SST climate change response (Fig. 7). In the past, the direct CO_2_ effect primarily contributed to the increase in precipitation. For future CO_2_ climate changes, the dryer signal over Pakistan is dominated by the SST effect, whereas the direct atmospheric response to CO_2_ changes alone would indicate a weak wet signal. The underlying dynamical upper tropospheric circulation components over Tibet are distinctly different for the two effects (see Fig. 8). Importantly though, it is the magnitude of their relative change that controls the total dynamical response, leading to the non-linear rainfall response signal over Pakistan.

Our approach applied spatially non-uniform perturbations to the SSTs and subsurface temperature and salinity at initial time, which were derived from observational estimates. Sensitivity experiments with idealised uniform temperature perturbations reduced the described limited impact in the rainfall totals due to CO_2_-induced climate change to even smaller signals. This raises the issue of how best to adjust the ocean initial conditions for the effects of climate change, and indeed there is clearly some uncertainty in estimating these effects. In future developments of this forecast-based attribution method, ensembles of perturbations for estimating the effects of climate change on initial conditions will be considered.

The results of this study are also relevant to the issue of climate adaptation and, on a broader scale, to the Sustainable Development Goals of the United Nations. Should Pakistan be investing in infrastructure to make its society more resilient to future flooding, or to future heat wave and drought? Results from our FUTURE ensembles suggest that overall rainfall may decrease. However, we also see a tendency that wet extremes might become more likely in the future climate, and hence Pakistan should indeed be prepared for both.

Primarily however, these results suggest that understanding how climate change affects monsoon regions in South Asia is not straightforward, contrary to what some media commentators suggested when reporting the Pakistan floods in 2022^2^. In particular, future climate change may affect essential circulation patterns like the upper tropospheric zonal flow over the Tibetan Plateau differently than it did in the past. While ECMWF’s seasonal forecasting system is remarkably realistic in its atmospheric circulation and rainfall characteristics, current CMIP-class climate models do not simulate these dynamical impacts, e.g., the local Walker- and Hadley circulation responses on the monsoon depressions, with any degree of reliability. Global climate projections keep showing an equivocal response of the corresponding circulation^33^ and large differences in the latest CMIP6 generation models are still apparent with the accurate representation of the prevailing physical processes remaining a challenge^5,6,34,35^. At the same time, biases against observations in current generation climate models are as large as the climate change signals they try to emulate^36^. Our results indicate that to guide investment in infrastructure to adapt to climate change, we need to develop and apply improved climate models with significantly reduced bias that are able to reliably simulate the relevant processes at the scales that matter^37,38^.

## Methods

### Seasonal forecast experiments

All seasonal experiments for this study are performed with ECMWF’s operational seasonal forecasting system SEAS5^14^. It is based on a dynamical coupled atmosphere-ocean-land-sea ice model where the atmosphere is run at a horizontal resolution of approximately 35 km with 91 levels in the vertical. The ocean is discretised at ¼ degree horizontally with equatorial refinement and 75 vertical levels. The forecasts are initialised on the 1^st^ May 2022 using ECMWF’s operational analyses of the atmosphere, land, ocean and sea-ice. Forecast anomalies are computed with respect to the SEAS5 hindcast climatology from retrospective forecasts started on each 1^st^ May during the period 1993–2016. The ensemble size of the forecasts and hindcasts is 51 members. Members of the operational SEAS5 ensemble are created through perturbations of both the initial conditions in the atmosphere and ocean as well as the physical tendencies in the atmospheric model throughout the integration.

The experiments preIND and FUTURE are performed specifically for this study: we apply changes to the CO_2_ concentration during the forecasts and perturbations to the ocean and sea-ice state at the start of the forecasts. The perturbations to the initial conditions reflect the adjustment of the global oceans to the decreased and increased CO_2_ concentrations, see details below. Experiments A and B use perturbed CO_2_ concentrations but the same initial conditions as SEAS5. All 4 experiments are performed with a similar ensemble size as the operation forecasts of SEAS5. The schematic in Fig. 6 illustrates the seasonal forecast experiments used in this study.

### Adjustment of the ocean initial conditions

The initial conditions for the preIND forecast are derived by subtracting perturbations of the 3D temperatures, sea-ice concentration and sea-ice thickness from the SEAS5 initial conditions; for the initial conditions of the FUTURE forecast these are added to the SEAS5 state. The perturbations emulate the estimated anthropogenic influence on the ocean state since the pre-industrial period and are computed through an optimal fingerprint analysis based on the Anthropogenic Warming Index (AWI)^39^ using anthropogenic and natural radiative forcings from AR6^39,40^ and the HadCRUT5 global surface temperature dataset^41^. Observed timeseries of sea-ice thickness and concentration (ORAS5^42^, 1958–2019), sea surface temperature (HadISSTv1.1^43^, 1870–2019) and subsurface temperature (WOA18 1950–2017) are then regressed, at each grid point, onto the AWI. The regression coefficients are scaled by the change in AWI between the pre-industrial period 1850–1900 and 2019 to produce our final perturbations. Sea surface and subsurface temperature perturbations are combined by relaxing the sea surface perturbations towards the subsurface perturbation using a relaxation depth scale of 60 m. After an adjustment through the equation of state of the ocean salinity such that the in-situ ocean density is preserved, the resulting coupled forecasts are thermodynamically consistent with the imposed ocean heat content anomalies without any adjustments to the initial ocean circulation, mixed layer depths, or horizontal pressure gradients. Importantly, and unlike uncoupled forecasts constrained by specific sea surface temperatures, there are no infinite sources or sinks of heat in the resulting counterfactual forecasts. We note that estimating the perturbations, and in particular the subsurface temperatures, is uncertain due to the lack of observations in the pre-ARGO era. Further details about this novel ocean adjustment are given in Leach et al. (2024)^20^.

**Fig. 10:**
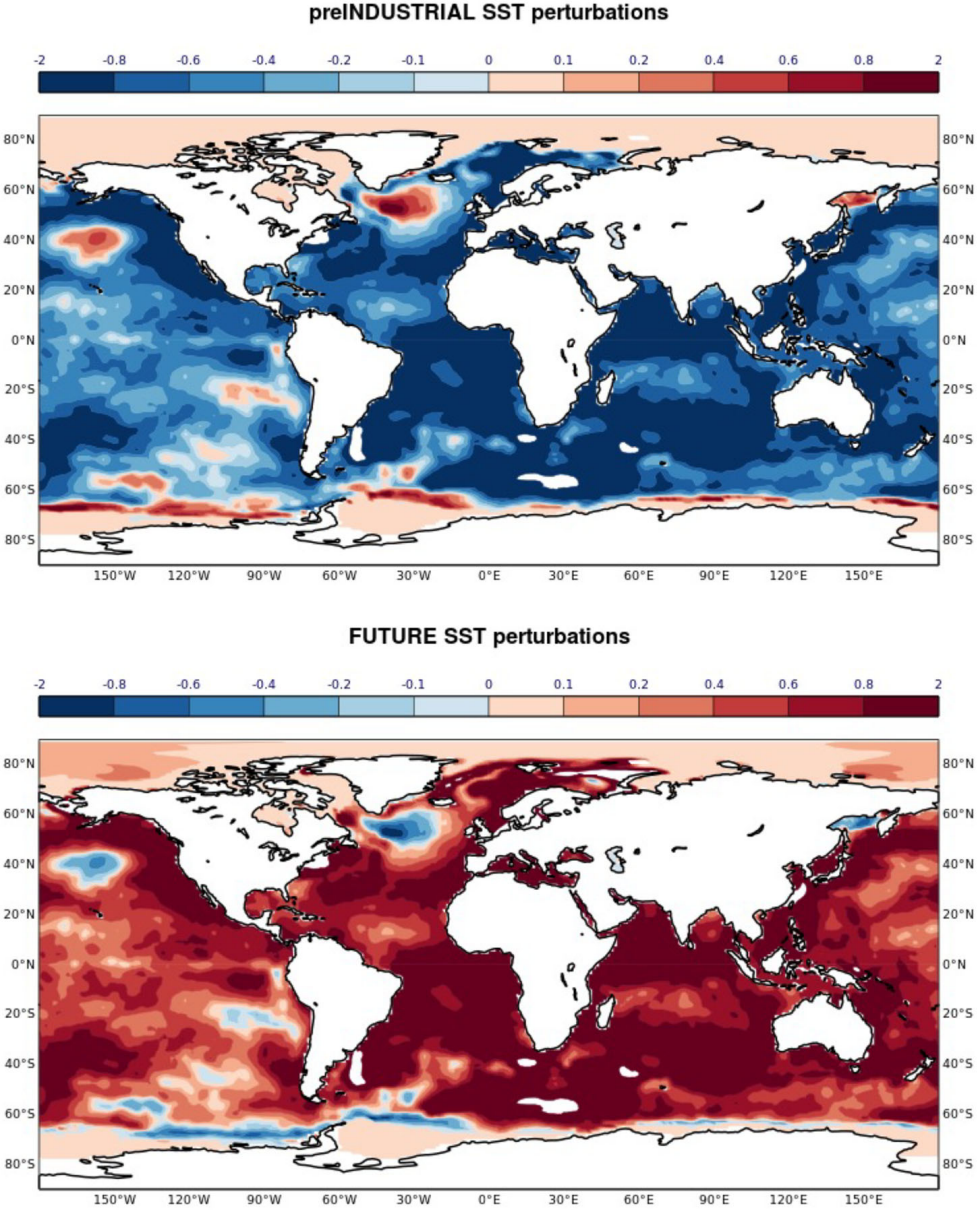
SST perturbations applied at initial time of the preIND and FUTURE experiments. The perturbations are symmetric by design. SST units: K.

Figure 10 shows the symmetric SST perturbations applied to the initial conditions of the preIND and FUTURE forecast experiments. Averaged over the Niño3.4 region of the equatorial Pacific (170°W-120°W, 5°S-5°N) as an index of ENSO, the perturbations amount to ±0.41 K which is approximately 90% of the SEAS5 anomaly of -0.47 K for the 2022 season. During the JJA season, the past climate change signal on the NINO3.4 index is +0.37 K which corresponds to an ~80% strengthening of the SEAS5 anomaly. The future signal amounts to +0.36 K which results in a weak cold La-Niña anomaly of -0.11 K (see Fig. 2d), implying that the future forcing conditions do not neutralise the cold anomalies in the equatorial Pacific. Positive SST anomalies are found though everywhere in the tropical Indian Ocean.

An additional set of idealised experiments were conducted with uniform SST and 3D ocean state perturbations. Here, the perturbations were constructed such that the global mean of the SST perturbations in the main experiments (Fig. 10) is conserved. While these experiments maintain the zonal gradient of SSTs across the tropical Pacific, very limited impact on seasonal mean SSTs and precipitation over Pakistan was found (not shown).

### Zonal flow index

Following He et al. (2023)^27^, we define an upper tropospheric zonal flow index over the region of the Tibetan Plateau where the climatological transition from tropical easterlies to extratropical westerlies occurs. The index is the average zonal flow anomaly at 200 hPa in the box 60°-120°E, 27°-33°N, as indicated by the cyan box in Figs. 5 and 8.

## Data Availability

ERA5 data and the seasonal forecasts from SEAS5 and other C3S contributors are accessible through the C3S Climate Data Store at https://cds.climate.copernicus.eu/cdsapp#!/home. Global data from the preIND and FUTURE forecast experiments used in this study are available through https://apps.ecmwf.int/research-experiments/expver/htk3/ and https://apps.ecmwf.int/research-experiments/expver/htkx/ under a Creative Commons Attribution 4.0 International license (CC BY 4.0). To view a copy of this license, visit https://creativecommons.org/licenses/by/4.0/.

## Code availability

The code of the coupled SEAS5 model is accessible only by ECMWF and its Member and Co-operating States. However, an open access version of the IFS code for the atmospheric and land components is available as part of the OpenIFS^44^.
